# A SNP in intron 8 of *CD46* causes a novel transcript associated with mastitis in Holsteins

**DOI:** 10.1186/1471-2164-15-630

**Published:** 2014-07-28

**Authors:** Xiuge Wang, Jifeng Zhong, Yundong Gao, Zhihua Ju, Jinming Huang

**Affiliations:** Dairy Cattle Research Center, Shandong Academy of Agricultural Sciences, No.159 North of Industry Road, Jinan, Shandong 250131 China; Division of Animal Sciences, University of Missouri, Columbia, MO 65211 USA

**Keywords:** *CD46*, Alternative splicing, Functional SNP, Cattle, Mini-gene system

## Abstract

**Background:**

The membrane protein CD46, a ubiquitous cell surface pathogen receptor, can bind *Streptococcus* to trigger cell autophagy, which is a critical step in the control of infection.

**Results:**

In this study, we found a new splice variant designated *CD46* transcript variant (*CD46-TV*). The splice variant is characterized by the retention of a 48 bp sequence from intron 8 of the bovine *CD46* gene, which encodes a putative protein enlarged by 16 amino acids. *CD46-TV* mRNA was found to be over expressed in mastitis-infected mammary gland tissues relative to healthy tissues. A single nucleotide polymorphism (c. 1033 + 2184 C > T) in the exonic splicing enhancer (ESE) motif region was shown to result in the *CD46-TV* aberrant splice variant through constructing alternative alleles using the pSPL3 exon capturing vector and transfecting these into 293 T cells. Allelic frequency in 56,682 individuals belonging to 112 *Bos taurus, Bos indicus, Bos javanicus, Bos grunniens and Bos mutus, etc.* suggests that the C allele (80.09%) is the ancestral allele. Association analysis found that the mean genomic estimated breeding values (gEBV) for milk somatic cell score and the occurrence of clinical mastitis, as well as the milk somatic cell score of Chinese Holsteins with the CT genotype was lower than those of individuals with either the CC or TT genotypes. The mean gEBV for udder health synthesis for the TT genotype was greater than those for the CC or CT genotypes.

**Conclusions:**

Our findings suggest that the *CD46* gene likely plays a critical role in the risk of mastitis caused by *Streptococcus* in dairy cows via an alternative splicing mechanism caused by a functional mutation in intron 8. Our data also underline the importance of variation within ESEs in regulating transcript processing.

**Electronic supplementary material:**

The online version of this article (doi:10.1186/1471-2164-15-630) contains supplementary material, which is available to authorized users.

## Background

The selection of susceptibility-related genes is considered to be one of the best long-term means to improve resistance to mastitis in dairy cattle and may reduce drug use, improve milk quality and safety, and reduce economic loss [1, 2]. As a prominent mechanism for posttranscriptional regulation, alternative splicing is significant in cell development, differentiation, physiological functions and pathogenesis, and it has been reported to have many critical roles in mammalian diseases including bovine mastitis [3–7]. Many studies have shown that alternative splicing is also closely related to immune function [8–10].

Pre-mRNA splicing involves a large molecular complex called the spliceosome that consists of four small nuclear ribonucleoprotein particles (U1, U2, U4/U6 and U5 snRNPs) and other non-snRNP splicing factors, such as serine/arginine rich (SR) and U2AF proteins [11]. The exons of vertebrate genes are generally separated by large introns, and possess 5’ splice sites, branch points, pyrimidine tracts and 3’ splice sites which are very short conserved elements that are required for constitutive splicing [12]. The splicing of alterative exons is also modulated by cis-acting elements, such as positive exon splicing enhancers (ESEs) which regulate the gene splicing process by interacting with SR proteins. Through binding to an ESE, SR proteins can enhance U1 snRNP binding to a 5’ splice site resulting in a splicing reaction, and can also promote U2AF recruitment to the pyrimidine tract, which leads to the activation of the adjacent 3’ splice site [13, 14]. As has previously been shown, mutations in ESEs result in aberrantly spliced transcripts, and may cause human diseases by affecting the encoded protein [15, 16].

CD46 is a single stranded transmembrane glycoprotein which anchors to almost all nucleated cells, including granulocytes, platelets, T cells, B cells, NK cells, fibroblast cells, skin cells, endothelial cells and stellate glial cells. CD46 protein is composed of a region comprising four structurally related short consensus repeats (SCRs), a serine, threonine and proline-rich (STP) region, a short region of unknown function, a hydrophobic transmembrane domain (TM) and a cytoplasmic tail (cyt). The STP region is variable, and encoded by three exons (A, B, C) which are commonly expressed as B + C or C isoforms. One of two non-homologous alternative cytoplasmic tails (cyt-1 or cyt-2) resulting from alternative splicing contains signaling motifs which drive the differential processing of isoforms [17, 18]. CD46 belongs to the family of regulators of complement activation, and cleaves complement factors C3b and C4b to protect normal cells from complement-mediated damage [19, 20]. CD46 can also be used as a costimulatory molecule involved in T cell activation through altering cell polarity and preventing normal cell responses to antigen presentation [21–23]. There are reports which show that pathogen receptor CD46 on the cell surface can trigger cell autophagy to control infection by *Streptococcus* [17, 24]. Bovine mastitis is the inflammation of the mammary gland caused primarily by bacteria, such as *Staphylococcus aureus, Streptococcus,* and *Escherichia coli.* Therefore, we speculated that the *CD46* gene likely plays a role in the defense of dairy cattle to *Streptococcus* induced mastitis by an alternative splicing mechanism caused by a functional mutation which causes an aberrant transcript.

We found that a SNP in intron 8 of the bovine *CD46* gene causes the partial retention of intron 8 sequences within mature *CD46* RNAs. By cloning alternative *CD46* partial alleles into a minigene expression system which was transfected and expressed in 293 T cells, we demonstrate that this mutation disrupts an ESE, and causes the production of an aberrant CD46-TV isoform. Association analysis between four udder health traits of Holsteins and their *CD46* SNP (c. 1033 + 2184 C > T) genotypes suggests that this SNP may be a useful functional marker for molecular breeding for mastitis-resistance in dairy cows.

## Results

### The novel splice variant *CD46-TV*was identified in mammary gland tissues

**Figure 1:**
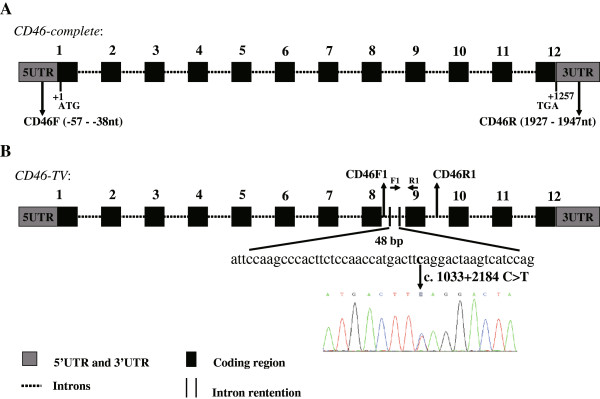
**Identification of a novel** ***CD46*** **splice variant. (A)** Genomic structure of the bovine *CD46* gene. *CD46* gene consists of 12 exons, comprising 1257 bp of coding sequence. The positions of the primers (CD46F, CD46R) used in the *CD46* cloning experiment are indicated by black arrows. **(B)** The splicing pattern of the *CD46-TV* splice variant. The *CD46-TV* (GenBank accession number: KM114056) transcript retains a 48 bp sequence from intron 8. The F1 and R1 primers are located within the 48 bp retained intron sequence and exon 9, respectively. The position of the A nucleotide in the start codon (ATG) is defined as +1.

Specific CD46F and CD46R primers were used to amplify the 5’ untranslated region (5’UTR) to the 3’ untranslated region (3’UTR) of the bovine *CD46* gene (Figure 1A) from the 4 pooled cDNA composed of 16 mammary tissue samples. PCR conditions for annealing were designed using 12 temperature gradients between 52°C and 65°C. In addition to the expected smaller 2005 bp PCR product, a larger band was also detected by electrophoresis on 2% agarose gel from the products produced at increased annealing temperatures (see Additional file 1: Figure S1), indicating that an aberrant splice variant might exist.

To confirm the putative bovine *CD46* splice variant, we purified and cloned the largest PCR product. A total of 30 clones were sequenced individually, and we identified the new splice variant, named as *CD46-TV* (GenBank accession number: KM114056), from 6 clones. Compared to the *CD46-complete* transcript, BLAST analysis indicated that the splice variant *CD46-TV* retained a 48 bp sequence from intron 8 (Figure 1B).

### A putative functional SNP in intron 8 of *CD46*

To identify the molecular cause of the aberrant *CD46-TV* splice variant, primers CD46F1 and CD46R1 were designed to amplify the adjacent region of intron 8 (Figure 1B). SNP (c. 1033 + 2184 C > T) was found by sequencing the PCR amplification products from dairy cow DNA, and is located in the 48 bp sequence retained in the *CD46-TV* transcript (Figure 1B).

**Figure 2:**
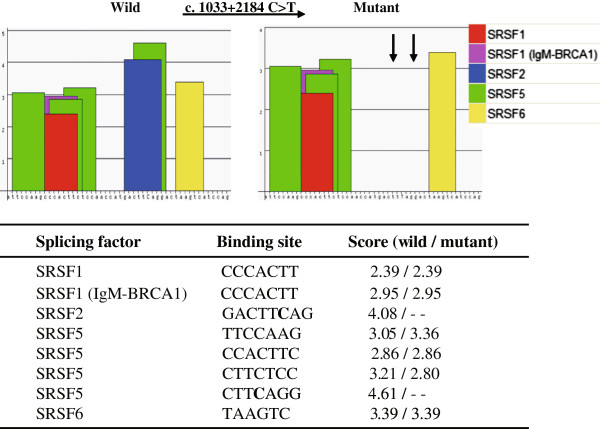
**Exonic splice enhancer (ESE) motif threshold scores associated with** ***CD46*** **genotypes.** Bar graphs represent scores above the threshold for the ESE motifs within the ancestral (wild) and mutant haplotypes. The arrows indicates that the signal for the SRSF2 and SRSF5 motifs disappear when the mutation (c. 1033 + 2184 C > T) is introduced into the wild-type sequence. Wild type (C) sequence: attccaagcccacttctccaaccatgactt**c**aggactaagtcatccag; Mutant type (T) sequence: attccaagcccacttctccaaccatgactt**t**aggactaagtcatccag.

ESEfinder 3.0 ( http://rulai.cshl.edu/cgi-bin/tools/ESE3/esefinder.cgi?process=home) [25] software was used to predict whether the SNP affected the production of the new transcript *CD46-TV*. ESEfinder predicted the SNP to be located within overlapping ESE motifs for SR proteins, and that the introduction of the c. 1033 + 2184 C > T mutation abolished two binding sites for the splicing factors SRSF2 (SC35) and SRSF5 (SRp40) (Figure 2). These analyses suggest that there should be a correlation between the SNP alleles and the presence or absence of the splice variant *CD46-TV*.

### The C allele of SNP (c. 1033 + 2184 C > T) is identified as the ancestral allele

Genotyping indicated that the C allele was more frequent in the sample of 230 Chinese Holstein cows (Table 1). To determinate which allele is the mutant, allele frequency was further investigated in diverged bovid species. Interestingly, the SNP (c. 1033 + 2184 C > T) is located on the Illumina BovineSNP50K Beadchip (SNP name: Hapmap48320-BTA-40139; rs ID: 41634826), thus we extracted genotype data for this SNP and estimated allele frequency in 56,682 individuals belonging to 112 breeds or species including *Bos taurus, Bos indicus, Bos javanicus, Bos grunniens and Bos mutus* (see Additional file 2: Table S1). Within *Bos taurus* breeds (e.g., Angus, Charolais, Hereford, Holstein, Jersey, Limousin and Simmental) the frequency of the T-allele was 0.2042 to 0.3495, while the T-allele was nearly absent in 12 other *Bos* species (see Additional file 2: Table S1) suggesting that C is the ancestral allele and T is the mutant.

**Table 1:** **Association analysis between different genotypes and somatic cell score in 230 Chinese Holstein cows**

Genotype	Sample number	Genotype frequencies	Allele frequencies	Somatic cell score
CC	102	0.44	0.71 (C)	4.91 ± 0.33^a^
CT	124	0.54		3.84 ± 0.35^b^
TT	4	0.02	0.29 (T)	4.97 ± 0.45^a^

Superscript different letters (a, b) in the same column denote difference *p* < 0.05.

### Mutation in the putative ESE generates the novel splice transcript

**Figure 3:**
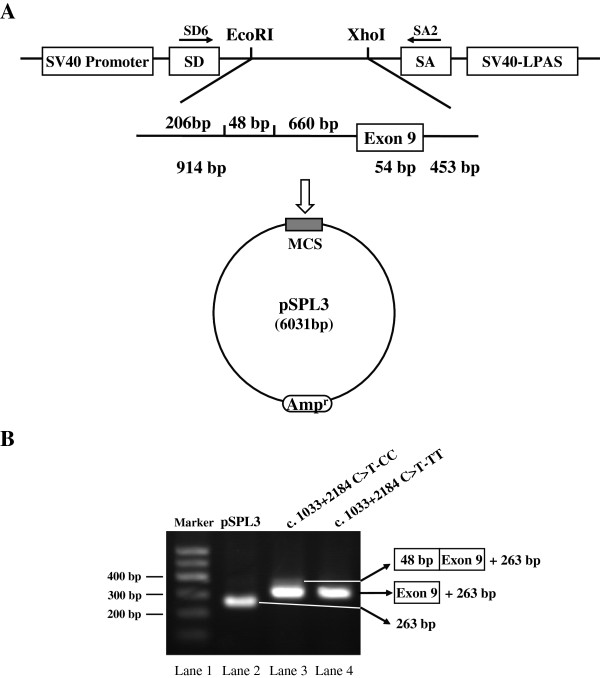
**The exon trapping vector pSPL3 used to assay SNP function. (A)** The pSPL3 vector contains SD (splice donor) and SA (splice acceptor) sites that operate as exons, and a functional intron, with transcription beginning following the SV40 promoter and ending at the LPAS (late poly **(A)** signal). Wild pSPL3-W and mutant pSPL3-M plasimds containing 914 bp of intron 8, exon 9 and 453 bp of intron 9 and harboring either the C or T alleles were separately cloned into the *Eco*RI and *Xho*I cloning sites of the pSPL3 vector. **(B)** Agarose gel electrophoresis of RT-PCR products. SD6 and SA2 primers were designed for RT-PCR amplification of cDNA sequences generated by transfected 293 T cells. Lane1: Marker;DNA Marker 600 (TIANGEN, China); Lane2: 263 bp; Lane3: 365 bp (263 bp + 54 bp + 48 bp) and 317 bp (263 bp + 54 bp); Lane 4: 317 bp (263 bp + 54 bp). MCS: multiple cloning sites.

To determine whether the c. 1033 + 2184 C > T nucleotide substitution in intron 8 leads to the abrogation of the predicted SR protein motifs and causes aberrant splicing, we respectively amplified 1421 bp genomic fragments spanning 914 bp of intron 8, 54 bp of exon 9 and 453 bp of intron 9 alternatively harboring the c. 1033 + 2184 C-allele and c. 1033 + 2184 T-allele and cloned the two fragments into the pSPL3 vector (Figure 3A).

After transfecting the two minigene plasmids into 293 T cells, mRNAs were isolated at 24 h following transfection, reverse transcribed and RT-PCR was applied to detect the *CD46* splicing pattern with vector-specific primers (SD6 and SA2). The wild-type and mutant-type constructs all gave rise to a 317 bp PCR fragment, and the empty pSPL3 control a 263 bp PCR product. However, the plasmid construct with the wild-type c. 1033 + 2184 C-allele also yielded two length of RT-PCR product. One is a larger RT-PCR product and the other is the 317 bp PCR band (Figure 3B). Through subsequent cloning experiment, we confirmed that the size of the larger band was 365 bp.

We also genotyped sixteen Chinese Holstein cows including 9 CC, 5 CT and 2 TT genotype individuals. Through RT-PCR experiment using F1 and R1 primers, the *CD46-TV* transcript was found to only be expressed in the mammary glands of cows possessing C allele. Further, the expression level of the *CD46-TV* transcript was relatively higher in cows with the CC than the CT genotype (see Additional file 1: Figure S2).

### The SR protein and ESE functions in the alternative splicing of CD46

**Figure 4:**
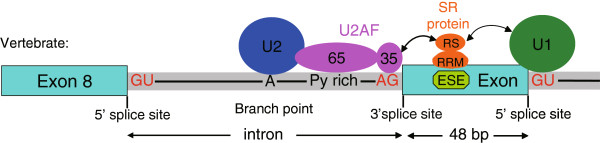
**SR protein and ESE functions for the bovine** ***CD46*** **gene.** The SR protein containing the serine-arginine residue (RS) domain and RNA recognition motif (RRM) is depicted as interacting with the exonic splicing enhancer (ESE). The SNP locates in the ESE. The U1 (green) and U2 (blue) spliceosomal snRNPs and the two small subunits of the U2AF auxilliary factors (U2AF65 and U2AF35) are required for ESE-dependent splicing.

Within the Metazoa, almost all of the pre-mRNA produced within the cell nucleus have GU bases in the 5’ splice site of adjacent introns and AG bases in the 3’ splice site, which is recognized as the GU-AG rule or Chambon rule [26]. To ascertain the reason for the retention of 48 bp of intron 8 sequence within the *CD46-TV* transcript, we analyzed the 48 bp sequence and the intronic splicing site bases, and found that the splicing site bases conformed with the GU-AG rule, while the c. 1033 + 2184 C > T mutation disrupted two ESE motifs recognized by the SRSF2 and SRSF5 proteins (Figure 4). We therefore infer that the presence of the SNP T allele eliminates the production of the *CD46-TV* splice variant.

### Analysis of *CD46-TV*mRNA and peptide sequences

**Figure 5:**
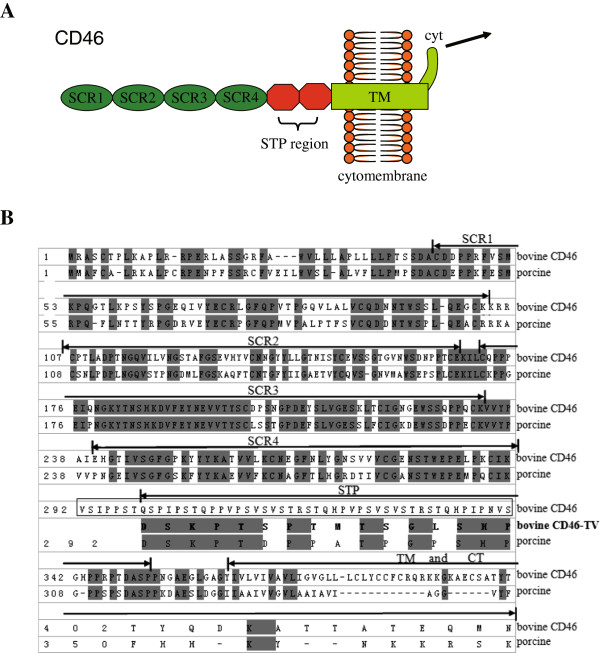
**Analysis of the** ***CD46*** **and** ***CD46-TV*** **transcripts. (A)** Diagram of *CD46* structure. The CD46 protein contains four short consensus repeats (SCRs), and a serine, threonine and proline-rich (STP) region that is followed by a short region of unknown function, a transmembrane domain (TM) and a cytoplasmic domain (cyt). The arrow indicates the signaling path after accepting the ligand. **(B)** Comparison of the bovine and porcine CD46 amino acid sequences. The bovine CD46 amino acid sequence (NCBI: NP_001229490.1) was aligned to the porcine sequence (NCBI: NP_999053.1) by BLAST. Identical amino acid residues within the two species are indicated in grey. The positions of the SCRs, STP, TM and CT (cyt) domains corresponding to different amino acids are shown above the sequences. Amino acids of the CD46-TV retention transcript in comparison to the porcine CD46 are bolded.

The identity of the bovine CD46, CD46-TV and other species (pig, dog, mouse, monkey and human) mRNA and peptide sequences is shown in Additional file 1: Figure S3 and Figure S4. A BLAST comparison of sequences from different species revealed that the bovine CD46 amino acid sequence (NCBI: NP_001229490.1) had a 59% identity with porcine CD46 (NCBI: NP_999053.1), 48% with human’ (NCBI: NP_002380.3) and 36% with mouse’ (NCBI: NP_034908.1) for the open reading frame shifting (see Additional file 1: Figure S3). From the analysis of the porcine CD46 amino acid, the sequences for the four SCR regions were identical. However, the STP, TM and cyt domains differed between the two species. Moreover, we found that the 16 amino acids translated from the 48 bp incorporated intronic sequence within the *CD46-TV* transcript were located in the STP region and were highly conserved with the porcine STP region (Figure 5 and see Additional file 1: Figure S3 and Figure S4).

### Expression of the *CD46-TV*splice variant in cow mammary gland tissues

**Figure 6:**
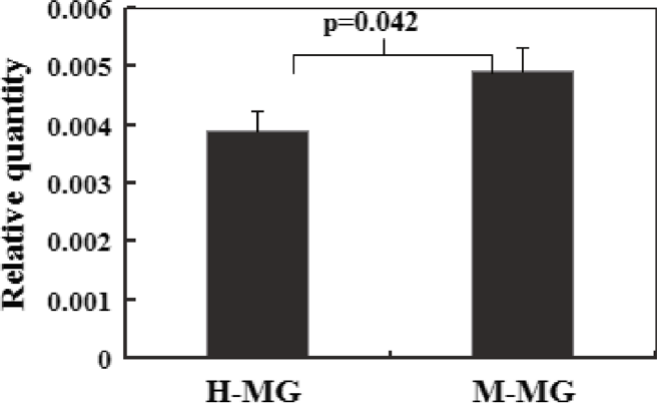
**Relative expression of the bovine** ***CD46-TV*** **transcript in different tissues.** Expression of the *CD46-TV* transcript was analyzed in different tissues by RT-qPCR using F1 and R1 primers. *β-actin* was used as the internal control. H-MG: Healthy mammary glands. M-MG: Mastitis-infected mammary glands. Expression of different tissue was triplicate.

To understand the relationship between the *CD46-TV* transcript and dairy cow mastitis, the relative quantification of the transcript in mammary glands was performed by RT-qPCR from three healthy and three *Streptococcus* infected and mastitic dairy cows with the CC genotype. The mastitis-infected tissues revealed a relatively higher *CD46-TV* mRNA expression compared with healthy tissues (p < 0.05, Figure 6), indicating that the C allele would be associated with a reduced risk of mastitis.

### Relationship between genotypes of the SNP (c. 1033 + 2184 C > T) and milk somatic cell score, as well as gEBV of STMA, CEL and MACL of Holsteins

Somatic cell count is considered to be an indicator for dairy mastitis [27] and it is often converted into somatic cell score (SCS) due to the skewness of the somatic cell count distribution [28]. To investigate whether the SNP was associated with mastitis in dairy cows, we first used the CD46F1 and CD46F1 primers to genotype 230 cows by direct sequencing, and the association analysis was performed between genotype and somatic cell score in these Chinese Holstein cows. The results revealed that the estimated frequencies of the C and T alleles were 71% and 29%, respectively. Moreover, cows with the CC and TT genotypes showed a relative higher SCS than individuals with the CT genotype (*p* < 0.05, Table 1).

CEL is the genomic breeding value of somatic cell score. MACL is an index used to evaluate the occurrence of clinical mastitis. gEBV for STMA is a combination of somatic cell score and clinical mastitis ( http://idele.fr/fileadmin/medias/Documents/INTRO_PH_141_va.pdf). A high breeding value means a high occurrence of mastitis. In experiment four, to investigate whether this SNP was associated with mastitis in Holsteins, we analyzed the association between genotypes and mastitis-related traits in 98 Chinese Holsteins with gEBVs. The estimated frequencies of the C- and T-alleles were 83.16% and 16.84%, respectively. Moreover, cows with the CC and TT genotypes showed a relative higher STMA and CEL than individuals with the CT genotype (*p* < 0.05, Table 2). The result suggests that there is some kind of heterozygote advantage although the mechanism by which this occurs is really not clear. Moreover, the gEBV of MACL of the Holstein with the CC or CT genotypes was significantly lower than for the TT genotype (Table 2). These results suggest that the CC and CT genotypes are advantageous for bovine mastitis resistance and we conclude that the c. 1033 + 2184 C > T mutation affects cow mastitis-resistance.

**Table 2:** **Genetic effect of SNP (c. 1033 + 2184 C > T) on gEBV of STMA, CEL and MACL traits in Holsteins**

Genotype	Sample number	Genotype frequencies	Allele frequencies	STMA gEBV	CEL gEBV	MACL gEBV
CC	68	0.69	0.8316(C)	0.1279^a^	0.2580^a^	−0.087^c^
CT	27	0.28		−0.0037^b^	0.1630^b^	−0.2500^b^
TT	3	0.03	0.1684 (T)	0.2330^a^	0.2333^a^	0.2^a^

gEBV - Genomic estimated breeding value; STMA - Udder health synthesis; CEL - Milk somatic cell score; MACL - Occurrence of clinical mastitis;

Superscript different letters (a, b, c) in the same column denote difference *p* < 0.05.

## Discussion

In this study, a new *CD46* transcript variant named *CD46-TV* was identified following the cloning of *CD46* mRNA. By sequencing, we identified the SNP (c. 1033 + 2184 C > T) within the retained 48 bp intron 8 sequence of *CD46*. Using the ESEfinder 3.0 bioinformatics tool, we predicted that this SNP was located within an exonic splice enhancer (ESE) motif, and that the derived T-allele eliminated two potential SR protein binding sites for the splicing factors SRSF2 and SRSF5. The SR proteins are highly conserved in metazoans, are required for constitutive splicing and can influence the regulation of alternative splicing. SR proteins function in the recognition of ESEs and lead to the activation of suboptimal adjacent 5’ and 3’ splice sites [14], and the SRSF2 (SC35) protein is responsible for the aberrant splicing of pre-mRNAs when increased numbers of binding sites result in the retention of intron and exon skipping [6, 29]. It has also been reported that the over-expression of SRSF5 (SRp40) affects the alternative splicing of *CD44* [30]. Therefore, the deletion of the two splicing factors may partially affect splicing which produced a product which retains the 48 bp intronic sequence.

To verify whether the c. 1033 + 2184 C > T mutation influenced the aberrant splicing of *CD46*, we introduced alternative alleles into the pSPL3 exon trapping vector and found that the pSPL3 construct vector with the C-allele permitted the retention of the 48 bp intron 8 sequence while the T-allele construct did not permit this retention. Alternative and constitutive splicing are closely connected and utilize the same splicing mechanism, because components of constitutive splicing are also essential for alternative splicing. Moreover, alternative exons often have suboptimal splice sites or sequence elements compared with constitutive exons, and different activities or amounts of splicing factors in different cell types or developmental stages can also affect the splicing reaction [11]. We analyzed the splicing position sequences producing the *CD46-TV* transcript, and found that the retained 48 bp sequence conformed to the conditions of an alternative exon and had the potential for alternative splicing. These results suggest that the disruption of the predicted SRSF2 and SRSF5 binding sites caused by the c. 1033 + 2184 C > T mutation leads to the retention of the 48 bp intron 8 sequence. CD46 regulates complement activation by binding the C3b and C4b complement products deposited on host cell membranes and serves as a cofactor for their proteolytic inactivation by the plasma serine protease factor I. This process, which irreversibly prevents C3 and C5 convertase formation, protects host cells from lysis by autologous complementation [19]. The reference CD46-complete protein and the putative CD46-TV protein share common SCR1-4 domains containing binding sites for C3b and C4b. Therefore, the two CD46 isoforms can play the same role in complement regulation. The mutation is predicted to increase the size of the CD46 protein by 16 amino acids. By BLAST analysis, we found that these 16 amino acids were highly conserved with the STP region of the porcine CD46 protein. The CD46 STP domain serves as a functional modulator of CD46 by altering complement regulatory activity [31]. The STP domain is also crucial for the efficient adhesion of bacteria to host cells [32, 33]. Recognition of microbial pathogens is an essential element for the initiation of innate immune responses such as inflammation. The complement regulatory protein CD46 is used as a cell receptor and can bind with bacteria including *Streptococcus pyogenes* [34]. Therefore, CD46-TV may play an important role in resistance to mastitis caused by *Streptococcus*.

To further demonstrate the function of *CD46-TV* in bovine mastitis, RT-qPCR demonstrated that *CD46-TV* mRNA expression was significantly higher in mastitis-infected mammary tissues than in healthy tissues. Autophagy is a degradative mechanism involved in cell protection against invading pathogens. CD46 can play a primary function in the host’s response to pathogens by regulating complement activation, and can also play an important role in CD46-dependent autophagy, which is induced as an innate immune mechanism and is a critical step in the early control of infection [35].

Several studies have investigated SNP associations with mastitis traits by genome-wide association studies in various cattle populations. Most of the reports highlighted the most significant SNPs and results of studies have differed. Many SNPs on different chromosomes (BTA 1, 3–6, 9–10, 13–17, 20–26 and X) have reached genome-wide significance for association with mastitis-related traits using the Illumina BovineSNP50 and BovineHD Beadchips [36–39]. Several SNPs with significant associations with clinical mastitis all lactations, somatic cell score and clinical mastitis first lactation were found on BTA16 ( http://onlinelibrary.wiley.com/store/10.1111/age.12053/asset/supinfo/age12053-sup-0003-FigS3.pdf?v=1&s=d93491c11600b5589883f66687870486940dff5e), however, detailed SNP information was not provided [39]. No specific evidence revealed association signals in the vicinity of CD46 on BTA16 were identified in these studies. This suggests that there may be larger effect mutations on mastitis susceptibility than the mutation which produces the *CD46-TV* transcript.

Recent evidence suggests that SNPs are major contributors to the generation of alternative splice variants, which can cause serious human and cow diseases [40, 41]. To test whether the c. 1033 + 2184 C > T SNP identified in this study plays an important role in dairy cow mastitis, an association analysis between genotypes and gEBVs of STMA, CEL and MACL showed that the Holsteins with the CT and CC genotypes were more resistant to mastitis because individuals with the TT genotype have a higher percentage of occurrence of clinical mastitis. Based on the results of the SNP causing the *CD46-TV* splice variant and the association analysis between genotype and gEBVs of mammary gland health traits in Chinese Holstein population, we predicted that CD46-TV protein caused by the SNP was very likely to have a significant role in bovine mastitis-resistance. This result requires further confirmation in a larger reference population.

## Conclusions

We have identified a causative mutation in an exonic splice enhancer of the bovine *CD46* gene which causes the elimination of the *CD46-TV* splice variant. Bioinformatic prediction, relative expression and association analysis between genotype and mammary gland health traits suggest that the splice variant may be involved in resistance to mastitis caused by *Streptococcus* in dairy cows.

## Methods

### Ethics statement

All experiments were carried out according to the Regulations for the Administration of Affairs Concerning Experimental Animals published by the Ministry of Science and Technology, China in 2004 and approved by the Animal Care and Use Committee from the Dairy Cattle Research Center, Shandong Academy of Agricultural Sciences, Shandong, P. R. China.

### Animal, tissue samples and dataset

Experiment one: Tissue samples were collected from healthy and mastitis-infected mammary glands of first lactation Chinese Holstein cows from a commercial slaughter plant in Jinan, Shandong Province, China. The cows were defined as healthy if they were without pathogen infection identified by a culture test and did not show any clinical signs (such as swelling, heat, redness, hardness or pain). Cows were diagnosed with clinical mastitis if the milk from one or more quarters of the mammary gland was abnormal in color or consistency, accompanied by heat, pain, redness, or swelling of the quarter. The mastitis-infected cows were collected following the presentation of clinical symptoms and *Streptococcus* identification via culture. A total of sixteen tissue samples (8 cases and 8 controls) were collected and quickly frozen in liquid nitrogen for DNA extraction for genotyping and RNA extraction for the identification of splice variants and characterization of the relative expression of *CD46-TV*.

Experiment two: To determinate which allele was the ancestral and which was the mutant allele, a total of 56,682 individuals belonging to 112 Bos taurus (i.e. Angus, Holstein, Hereford, Limousin, Simmental, Luxi, etc.), or, Bos indicus (i.e., Boran, Brahman) breeds, and the buffalo (i.e., Cape buffalo), yak and bison species were used to analyze allele frequency at the SNP. Detailed information of animal samples is shown in Additional file 2: Table S1. These animals were genotyped with the Illumina BovineSNP50K Beadchip (San Diego, CA). The SNP (c. 1033 + 2184 C > T) data was extracted from the BovineSNP50 data (Marker name of the SNP: Hapmap48320-BTA-40139; rs ID: 41634826).

Experiment three: A total of 230 Chinese Holstein cows, 3–5 years old, from the standardized dairy cattle farms (Shandong, China) were selected randomly for estimation of the genetic effect of the SNP on somatic cell score. All animals were participated enrolled in the Dairy Improvement Herd testing and were milked three times daily for a month for the data used in the association study. The somatic cell count of milk samples was measured by using a Fossomatic Cell Counter (Foss Electric, FOSSMATIC 5000, Denmark). The blood of animals was collected from the jugular vein and was placed in a tube with acid-citrate-dextrose anticoagulant for genomic DNA extraction using the Tianamp Genomic Extraction Kit (Tiangen, Beijing, China). DNA concentration was estimated spectrophotometrically, and was diluted to 50 ng/mL. DNA samples were stored at −20°C for subsequent analysis.

Experiment four: A total of 60 Chinese Holstein cows and 38 bulls that originated from various male lineages from the Shandong OX Biotechnology Ltd and with genomic estimated breeding values (gEBVs) for udder health synthesis (STMA), milk somatic cell score (CEL) and occurrence of clinical mastitis (MACL) were used to analyze the association between SNP genotypes and gEBV for STMA, CEL and MACL. The gEBV for Holstein udder traits was computed by the Animal Genetics Department of INRA (National Institute for Agronomic Research) and gEBV data were provided by Genes Diffusion (Douai Cedex, France).

### Cloning of the bovine *CD46*gene coding region

Total RNA was isolated using the RNAsimple total RNA Kit (Tiangen, Beijing, China) from the mammary tissues and 2 μg RNA was reverse-transcribed to cDNA using RevertAid First Strand cDNA Synthesis Kit (Fermentas, Pittsburgh, PA, USA) according to the manufacturer’s instructions. The specific primer pair (CD46F: 5′-TGGACTCAGCAAGGTCTCTG-3′, CD46R: 5′-GCCATGTTGACCTACCCCATA-3′; Product size = 2005 bp) was designed to amplify the coding region of the *CD46* gene according to the *CD46* mRNA reference sequence (GenBank. NM_001242561.1). The reaction system for PCR amplification of the *CD46* cDNA was described in a previous report [6]. Conditions for PCR were 4 min at 94°C, followed by 35 cycles of 94°C for 30 s, between 52°C and 65°C designed 12 temperature gradients for 30 s, and 72°C for 1.4 min, and a final 72°C extension for 5 min. PCR products were purified, and cloned into the pEASY–T3 Vector (TransGen, Beijing, China).

### Relative quantitative analysis of *CD46*transcripts

Real-time quantitative PCR (RT-qPCR) was performed to investigate the expression of the splice variant *CD46-TV* in healthy and mastitis-infected dairy cow tissues. The relative expression level of the different tissues was normalized using the housekeeping gene *β-actin*. The RT-qPCR primers for specific *CD46-TV* and *β-actin* were as follows: F1: 5′-GCCCACTTCTCCAACCATGA-3′ (The primer sequence locates in the retained intronic seqence), R1: 5′-AAACCCTCAGCACCGTTAGG-3′, Product size = 93 bp; β-actinF: 5′-GCACAATGAAGATCAAGATCATC-3′, β-actinR: 5′-CTAACAGTCCGCCTAGAAGCA-3′; Product size = 173 bp. The RT-qPCR reaction was performed under the following conditions: 94°C for 5 min; 40 cycles at 94°C for 15 s; 60°C for 5 s. The detailed RT-qPCR protocol was previously described [6].

### Mutation screening

PCR primers (CD46F1: 5′-CCCGTGCAACATTCTTGTGC-3′, CD46R1: 5′-TAAGTG TGCTACCCCACCCT-3′) were designed from the *CD46* gene sequence (NCBI Reference Sequence: NC_007314.4) to amplify the region around the retention position of the intron 8 sequence in the *CD46-TV* transcript to screen for potential SNP. PCR products from 16 individuals were sent to BGI (Beijing, China) for direct sequencing using an ABI 3730xl instrument.

### Minigene constructs

A 1421 bp genomic segment of *CD46* spanning exon 9, 914 bp of intron 8 and 453 bp of intron 9 flanking sequences was amplified from genomic DNA of heterozygotes and then the fragments with the wild or mutant alleles that were identified by sequencing were cloned into the exon trapping vector pSPL3 (Invitrogen, CA, USA), using specific primers linking the *Eco*RI and *Xho*I restriction enzyme sites (CD46-F2: 5′-*CCGGAATTC*CCCGTGCAACATTCTTGTGC-3′, *CCGGAATTC*: *Eco*RI; CD46-R2: 5′-*CCGCTCGAG*TAAGTGTGCTACCCCACCCT-3′, *CCGCTCGAG*: *Xho*I). The ancestral and mutant type constructs were named pSPL3-W and pSPL3-M, respectively. All constructs were verified to contain the correct sequence by direct sequencing.

### Cell culture and transfection

Human epithelial kidney 293 T (HEK 293 T) cells were cultured in DMEM medium containing 10% fetal bovine serum (FBS), penicillin (100 U/L), and streptomycin (100 mg/L) at 37°C in a 5% CO_2_ atmosphere. One day before transfection, cells were transferred to 6-well culture plate to grow to approximately 70% to 80% confluence in an antibiotic free medium. Cells were then transfected with 4 μg plasmid DNA (pSPL3-W, pSPL3-M and empty pSPL3-control each) using OPTI-MEM® I Medium and Lipofectamine 2000 (Invitrogen, Carlsbad, CA, USA) according to the manufacturer’s instructions. Cells were harvested and total RNA was extracted after 24 h transfection with the RNAsimple Total RNA Kit (Tiangen, Beijing, China).

### Minigene expression analysis

A total of 1 μg of RNA from each of the transfected cells was reverse transcribed into cDNA using the SA2 primer (5'-ATCTCAGTGGTATTTGTGAGC-3') which is specific for the amplification of the pSPL3 vector and the RevertAid™ First Strand cDNA Synthesis Kit (Fermentas, Pittsburgh, PA, USA) according to the manufacturer’s specifications.

The cDNA was amplified with pSPL3 vector specific primers (SD6: 5'-TCTGAGTCACCTGGACAACC-3'; SA2: 5'-ATCTCAGTGGTATTTGTGAGC-3'). The PCR amplification conditions were as follows: an initial denaturation at 94°C for 4 min, followed by 30 cycles at 94°C for 30 s, 58°C for 30 s, 72°C for 2 min, and a final extension at 72°C for 5 min. PCR products were analyzed by electrophoresis on a 2% agarose gel.

### Statistical analysis

The association between the *CD46* SNP marker genotypes and somatic cell score was analyzed by the least squares method as applied in the general linear models (GLM) procedure of SAS9.0 (SAS Institute Inc, Cary, NC, USA). The GLM was as follows:


Where Y_*ijklmn*_ was the observed somatic cell score value; μ was the overall mean; F_*i*_ was the fixed effect of farm; G_*j*_ was the fixed effect of genotype; S_*k*_ was the fixed effect of sire; E_*l*_ was the fixed effect of season; H_*m*_ was the fixed effect of parity; e_*ijklmn*_ was the random residual effect.

The association between the genotypes and gEBV for STMA, CEL and MACL of bulls was tested using the T-test and One-way ANOVA program using the SAS 9.0 software. Values of *p* < 0.05 were considered significant.

## Electronic supplementary material

Additional file 1: **Figure S1.** RT-PCR products from the *CD46* gene expressed in bovine mammary tissues. **Figure S2.** Association between *CD46* genotype and *CD46-TV* transcript abundance. **Figure S3.** Comparisons of bovine *CD46-TV* and other species’ CD46 mRNA sequences. **Figure S4.** Comparisons of bovine CD46-TV and other species’ CD46 amino acid sequences. (DOCX 131 KB)

Additional file 2: **Table S1.** Genetic parameters of the SNP (c. 1033 + 2184 C > T) in 112 species. (XLSX 21 KB)
