# Supplementary material for: A SNP in intron 8 of CD46 causes a novel transcript associated with mastitis in Holsteins
Source: BMC Genomics. 2014 Jul 28;15(1):630. doi: 10.1186/1471-2164-15-630 (PMC4124149; doi:10.1186/1471-2164-15-630)
Supplement: Supplementary file 1 — Additional file 1: Figure S1. RT-PCR products from the CD46 gene expressed in bovine mammary tissues. Figure S2. Association between CD46 genotype and CD46-TV transcript abundance. Figure S3. Comparisons of bovine CD46-TV and other species’ CD46 mRNA sequences. Figure S4. Comparisons of bovine CD46-TV and other species’ CD46 amino acid sequences. (DOCX 131 KB) [file 12864_2013_6328_MOESM1_ESM.docx]

**Supplementary Figures**

**Figure S1 RT-PCR products from the *CD46* gene expressed in bovine mammary tissues.** The smaller band is the expected 2005 bp PCR product. The arrow indicates the samples for which annealing temperature increased during RT-PCR. Triangle indicated the larger band. Marker: DNA Marker 2000 (TAKARA, China).


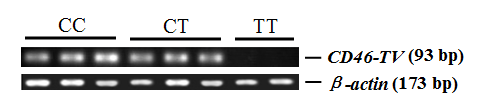


**Figure S2 Association between *CD46* genotype and *CD46-TV* transcript abundance.** Expression of the *CD46-TV* transcript was found only in individuals with the CC and CT genotypes.

**Figure S3 Comparisons of bovine *CD46-TV* and other species’ *CD46*** **mRNA sequences.**

Bos_taurus (NM_001242561.1); Sus_scrofa (NM_213888.1); human (NM_002389.4); Macaca_mulatta (NM_001195746.1); Mus_musculus(NM_010778.3); Canis_lupus_familiaris (XM_005622321.1)

7 1394

Bos_taurus.seq ------ATGA GGGCGTCTTG CACCCCGCTG AAGGCGCCGC TCCGCCGCCC

Bos_taurus_CD46-TV.seq ------ATGA GGGCGTCTTG CACCCCGCTG AAGGCGCCGC TCCGCCGCCC

Sus_scrofa.seq ------ATGA TGGCGTTTTG CGCGCTGCGC AAGGCACTTC CCTGCCGTCC

human.seq ---------- ---------- ---------- ATGGAGCCTC CCGGCCGCCG

Macaca_mulatta.seq ---------- ---------- ---------- ATGGCGCCTC CCGGCCGCCG

Mus_musculus.seq ATGACGGCGG CGCCTCTTAT GCCAGACTCA ACGCACCCCT GTCGCAGAAG

Canis_lupus_familiaris.seq ------ATGA CGGCGTCCCG CGCACCGCGC ACCCGCGGTC CCTGCTGCCC

CGAAAGACTG GCTTCTTCTG GGCGCTTCGC CTGGGTGCTT CTGC------

CGAAAGACTG GCTTCTTCTG GGCGCTTCGC CTGGGTGCTT CTGC------

CGAGAATCCC TTTTCTTCGA GGTGCTTCGT TGAGATTCTT TGGGTGTCGT

CGAGTGTCCC TTTCCTTCCT GGCGCTTTCC TGGGTTGCTT CTGG------

CGAGCGTCCC TTTTCTTCCG GTCGCTTTCC TGGGTTGCTT CTGG------

GAAGAGCTAC ACTTTCTTCT GGTGCTCCTT GGGCGTCTAT GCTG------

CCTGAGCCCC TCCTGTTCGC CGCGCTGCTC TCAGCCTCTC CGGG------

TGGCGCCGCT GCTCCTGCTG CCCACGTCCT CCGATGCCTG TGA---TGAT

TGGCGCCGCT GCTCCTGCTG CCCACGTCCT CCGATGCCTG TGA---TGAT

TGGCCCTAGT GTTCCTGCTT CCCATGCCCT CAGATGCCTG TGA---TGAG

CGGCCATGGT GTTGCTGCTG TACTCCTTCT CCGATGCCTG TGA---GGAG

CTACCCTCGT GTTGCAGCTA TCCTCCTTCT CCGATGCCTG TGA---GGCG

AGGCCCTTCT GTTTCTGCTG TCTCATTTAT CTGATGCCTG TGA---ACTA

GCTTCCTAAT GCTCCTCCTG CTACACTCCT GGGTTGTGGA TGCCTGTGAT

CCACCAAGAT TTGTCTCTAT GAAGCCCCAG GGTACCCTTA AACCCAGTTA

CCACCAAGAT TTGTCTCTAT GAAGCCCCAG GGTACCCTTA AACCCAGTTA

CCACCGAAGT TTGAAAGCAT GCGGCCCCAA TTTTTG---A ATACCACTTA

CCACCAACAT TTGAAGCTAT GGAGCTCATT GGTAAACCAA AACCCTACTA

CCACCAACAT TTGAAGCTAT GGAGCTCATT GGTAAACCAA AACCCTACTA

CCACGGCCAT TTGAAGCTAT GGAACTCAAG GGTACACCTA AACTCTTTTA

CGTCCAGCAT ACATCTCTAT GAAGCCAAAT GTTAGC---A AAATGAACTT

TAGTCCTGGG GAGCAGATTG TGTATGAATG TCGTCTGGGT TTCCAGCCAG

TAGTCCTGGG GAGCAGATTG TGTATGAATG TCGTCTGGGT TTCCAGCCAG

CAGACCTGGA GACCGTGTAG AGTATGAATG TCGCCCCGGG TTCCAGCCCA

TGAGATTGGT GAACGAGTAG ATTATAAGTG TAAAAAAGGA TACTTCTATA

TAGGGTTGGG GAACGAGTAG ATTATAAGTG TAAAAAAGGA TACTTCTATA

TGCCGTTGGA GAGAAAATAG AATATAAGTG TAAAAAAGGA TACCTGTATC

TGACCCTGGG GACACGATAT TCTTTACCTG TAATCTAGGA TACAGGCCTA

TAACTCCTGG TCAAGTCCTG GCTCTCGTTT GTCAGGATAA TAATACATGG

TAACTCCTGG TCAAGTCCTG GCTCTCGTTT GTCAGGATAA TAATACATGG

TGGTTCCTGC GCTTCCCACC TTTTCCGTCT GTCAGGACGA TAATACGTGG

TACCTCCTCT TGCCACCCAT ACTATTTGTG ATCGGAATCA TACATGGCTA

TACCTCCTCT TGCCACCCAT ACTATTTGTG ATCGGAATCA TACATGGCTA

TGTCTCCATA CCTGATGATT GCTACCTGTG AGCCAAATCA TACATGGGTC

TTAGGCCTCT CCTTCCCATG TCTTCTGTTT GTCAGCCTGA TAATACATGG

TCGTCTCTCC AGGAGGGCTG TAAAAAAAGA CGGTGTCCTA CCCTAGCTGA

TCGTCTCTCC AGGAGGGCTG TAAAAAAAGA CGGTGTCCTA CCCTAGCTGA

TCACCCCTCC AGGAGGCTTG TCGACGAAAA GCCTGTTCGA ATCTACCAGA

CCTGTCTCAG ATGACGCCTG TTATAGAGAA ACATGTCCAT ATATACGGGA

CCTGTCTCAG ATGAGGGCTG TTATAGAGAA ATGTGTCCAC ATATACGGGA

CCTATTTCAG ATGCTGGTTG TATTAAAGTT CAATGTACTA TGTTACAAGA

ACCCCTCTCA AGGAGGGTTG TACAAAAAAA TCATGTCTAC ATCCAGGAGA

TCCCACAAAT GGCCAAGTTA TCCTTGTAAA TGGAAGTACT GCGTTTGGCT

TCCCACAAAT GGCCAAGTTA TCCTTGTAAA TGGAAGTACT GCGTTTGGCT

CCCGTTAAAT GGCCAAGTTA GCTACCCAAA TGGGGATATG CTGTTTGGTT

TCCTTTAAAT GGCCAAGCAG TCCCTGCAAA TGGGACTTAC GAGTTTGGTT

TCCTTTAAAT GGTGAAGCAA TCCTTGCAAA TGGGTCTTAC GAGTTTGGTG

CCCTTCATTT GGCAAAGTAT ACTACATAGA TGGCAGCTTT TCATGGGGTG

ACCCAGCAAT GGCCAGGTAG TCCTCGTAGA TGAATCTCTG CTCTTTGGTT

CAGAGGTTCA CTATGTTTGT AATAATGGTT ATTACTTACT GGGGACAAAT

CAGAGGTTCA CTATGTTTGT AATAATGGTT ATTACTTACT GGGGACAAAT

CAAAGGCTCA GTTTACCTGT AACACTGGTT TTTACATAAT TGGAGCCGAG

ATCAGATGCA CTTTATTTGT AATGAGGGTT ATTACTTAAT TGGTGAAGAA

CTGAGTTACA CTTTATTTGT AACGAGGGTT ATTACTTAAT TGGTAAAGAT

CTCGAGCTAA ATTTACTTGT ATGGAAGGTT ATTACGTAGT TGGTATGTCA

CAAAGATTCA ATATTCCTGT AATGAGGGTT TTCGGTTAGT TGGACAAAAA

ATTTCTTATT GTGAAGTTTC TTCTGGAACT GG---T---- --GTGAACTG

ATTTCTTATT GTGAAGTTTC TTCTGGAACT GG---T---- --GTGAACTG

ACTGTGTATT GTCAGGT--- TTCTGGGAAT GT---T---- --ATGGCCTG

ATTCTATATT GTGAACTTAA AGGA---TCA GT---A---- --GCAATTTG

ATTCTATATT GTGAACTTAA AGAC---ACA GT---A---- --GCAATTTG

GTTCTACACT GTGTGCTTAA AGGT---GAT GATGAA---- --GCATACTG

AATCTATACT GTGAAATTTC TAGTACTGAC AG---TAATA GAGTGGTTTG

GAGTGATAAT CCTCCAACAT GTGAAAAGAT TTTGTGTCAA CCGCCTCCAG

GAGTGATAAT CCTCCAACAT GTGAAAAGAT TTTGTGTCAA CCGCCTCCAG

GAGTGAGCCC TCCCCGCTAT GTGAGAAGAT TTTGTGTAAA CCACCTGGCG

GAGCGGTAAG CCCCCAATAT GTGAAAAGGT TTTGTGTACA CCACCTCCAA

GAGCGGTAAG CCCCCATTAT GTGAAAAGAT TTTGTGTACA CCACCTCCAA

GAATGGCTAT CCCCCACATT GTGAAAAGAT TTATTGTTTA CCACCTCCAA

GAGTGATGAT CCCCCACTGT GTACAAAGAT ACTGTGTCAG CCACCTGGAA

AAATTCAAAA TGGAAAATAC ACCAATAGCC ACAAGGATGT ATTTGAATAC

AAATTCAAAA TGGAAAATAC ACCAATAGCC ACAAGGATGT ATTTGAATAC

AAATTCCAAA TGGAAAATAC ACCAATAGCC ATAAGGATGT ATTTGAATAC

AAATAAAAAA TGGAAAACAC ACCTTTAGTG AAGTAGAAGT ATTTGAGTAT

AAATAAAAAA TGGAAAACAC ACCTTTAGTG AAGTAGAAGT GTTTGAGTAT

AAATAAAAAA TGGAACACAT ACCCTTACTG ATATAAATGT ATTCAAATAC

AAATAGAAAA TGGAAAATAT TCTGATAGCC ACAAGGATGA ATTTGAATAT

AATGAAGTAG TAACTTATAG TTGTGATCCT TCAAATGGGC CAGATGAATA

AATGAAGTAG TAACTTATAG TTGTGATCCT TCAAATGGGC CAGATGAATA

AATGAAGTAG TAACTTACAG TTGTCTTTCT TCAACTGGAC CGGATGAATT

CTTGATGCAG TAACTTATAG TTGTGATCCT GCACCTGGAC CAGATCCATT

CTTGATGCAG TAACTTACAG TTGTGATCCT GCACCTGGAC CAGATCCATT

CATGAAGCAG TAAGTTACAG TTGTGATCCT ACCCCAGGGC CAGATAAGTT

AATGAAGTGG TAACTTACAG CTGTGAAAAG TCACAAGGAA CAGATGAATA

TTCCCTTGTT GGAGAGAGCA AGCTTACTTG TATTGGAAAT GGTGAATGGA

TTCCCTTGTT GGAGAGAGCA AGCTTACTTG TATTGGAAAT GGTGAATGGA

TTCACTTGTT GGAGAGAGCA GCCTTTTTTG TATTGGGAAG GACGAGTGGA

TTCACTTATT GGAGAGAGCA CGATTTATTG TGGTGACAAT TCAGTGTGGA

TTCACTTATT GGAGAGAGCA TGATTTATTG TGGTAACAAT TCAACATGGA

TTCCCTTGTT GGAACAAGCA TGATATTCTG TGCTGGCCAT AACACCTGGA

TTCACTTATT GGAGACAATA AGCTTATTTG TTCTGGCGAT GGTGAATGGA

GTAGTCAACC CCCTCAGTGT AAAGTGGTCA AATGTGTATA TCCAGCCATT

GTAGTCAACC CCCTCAGTGT AAAGTGGTCA AATGTGTATA TCCAGCCATT

GTAGTGACCC CCCTGAGTGT AAAGTGGTCA AATGTCCATA TCCAGTAGTC

GTCGTGCTGC TCCAGAGTGT AAAGTGGTCA AATGTCGATT TCCAGTAGTC

GTCATGCTGC TCCAGAGTGT AAAGTGGTCA AATGTCGATT TCCAGTAGTC

GTAACAGCCC TCCGGAGTGT AAAGTGGTAA AATGTCCAAA TCCAGTACTA

GTAGTAACCC TCCTGAGTGT AAAGTGGTCA GATGTCCACT TCCAGACCCC

GAACATGGAA CGATAGTCTC AGGATTTGGA CCAAAATATT ACTACAAAGC

GAACATGGAA CGATAGTCTC AGGATTTGGA CCAAAATATT ACTACAAAGC

CCAAATGGAG AAATTGTATC AGGATTTGGA TCAAAATTTT ACTACAAAGC

GAAAATGGAA AACAGATATC AGGATTTGGA AAAAAATTTT ACTACAAAGC

GAAAATGGAA AACAGATATC AGGATTTGGA AAAAAATTTT ACTACAAAGC

CAAAATGGAA GACTGATATC AGGAGCTGGC GAAATATTTT CCTATCAATC

GAAAATGGCA AACTGGTGTT GGGATTTAGC AGGAAATACT ACTACAAAGC

GACGGTTGTA CTTAAATGCA ATGAGGGTTT TAACCTTTAT GGCAACAGTG

GACGGTTGTA CTTAAATGCA ATGAGGGTTT TAACCTTTAT GGCAACAGTG

AGAGGTTGTA TTTAAATGCA ATGCTGGTTT TACCCTTCAT GGCAGAGACA

AACAGTTATG TTTGAATGCG ATAAGGGTTT TTACCTCGAT GGCAGCGACA

GACAGTTATG TTTGAATGCG ATAAGGGTTA TTACCTCAAC GGCAGCGACA

AACAGTGATG TTTGAGTGTT TGCAAGGATT TTACATGGAA GGCAGTAGCA

AAGAATTGAA TTTGAATGCC TTTCAGGGTT TTACCATAAG GGTACCAATT

TAGTTGTCTG TGGTGAGAAC AGTACTTGGG AGCCCGAGCT ACCAAAGTGT

TAGTTGTCTG TGGTGAGAAC AGTACTTGGG AGCCCGAGCT ACCAAAGTGT

CAATTGTCTG CGGTGCAAAC AGCACGTGGG AGCCTGAGAT GCCCCAATGT

CAATTGTCTG TGACAGTAAC AGTACTTGGG ATCCCCCAGT TCCAAAGTGT

AAATTGTCTG TGAGAGTAAC AGTACTTGGG ATCCCCCAGT TCCAAAATGT

TGGTGATCTG TAGTGCTAAT AACTCTTGGG AGCCATCTAT CCCAAAATGT

TTGCAATCTG TGGCAGTAAC AGTACTTGGG AGCCTGAGAT GCCGATGTGT

ATTAA----- ---------- ---------- ---------- AGTGTCGATT

ATTAA----- ---------- ---------- ---------- AGTGTCGATT

ATCAA----- ---------- ---------- ---------- ----------

CTTAAAGTGC TGCCTCCATC TAGTACAAAA CCTCCAGCTT TGAGTCATTC

CTTAA----- ---------- ---------- ---------- ----------

CTTAAAGGTC CTAGG----- ---------- ---------- ----------

CTTAA----- ---------- ---------- ---------- AGTGCCGATT

CCTCCCAGCA CCCAGTCTCC AAT------- ---------- -------TCC

CCTCCCAGCA CCCAGTCTCC AAT------- ---------- -------TCC

---------- ---------- ---------- ---------- ----------

AGTGTCGACT TCTTCCACTA CAAAATCTCC AGCGTCCAGT GCC-------

AGTGTCGACT TCTCCCACTA CAAAATCTCC AACGTCCAGT GCC-------

---------- ---------- ---------- ---------- ----------

CCTCCTACTA CCAGTCCTCC GATTTTGAGT CATACAGTGT CGAGTCCTCC

CAGTACCCAA CCTCCAGTTC CCAGTGTCTC AGTGTCGACT CGCAGCACCC

CAGTACCCAA CCTCCAGTTC CCAGTGTCTC AGTGTCGACT CGCAGCACCC

---------- ---------- ---------- ---------- ----------

---------- ---------- ---------- ---------- ----------

---------- ---------- ---------- ---------- ----------

---------- ---------- ---------- ---------- ----------

CAGTACAAAC TCTCCAATTC CGAGTGTCTC AGGATCT--- ----------

AACATCCAGT TCCCAGTGTC TCAGTGTCGA CTCGCAGCAC CCAACATCCA

AACATCCAGT TCCCAGTGTC TCAGTGTCGA CTCGCAGCAC CCAACATCCA

---------- ---------- ---------- ---------- ----------

---------- ---------- ---------- ---------- ----------

---------- ---------- ---------- ---------- ----------

---------- ---------- ---------- ---------- ----------

---------- ---------- ---------- ---------- ----------

ATTCCCAATG TCT------- ---------- ---------- ----------

ATTCCCAATG TCTCAGATTC CAAGCCCACT TCTCCAACCA TGACTTCAGG

---------- ----AGATTC CAAGCCTACT GATCCACCTG CAACCCCAGG

---------- --------TC AGGTCCTAGG CCTACTTACA AGCCTCCAGT

---------- --------TC AGGTCCTAGG CCTACTTACA AGCCTCCAGT

---------- ---------- ----CCTACT CATCCTACCA AGCCTCCAGT

---------- ---------- ---------- ---------- ----------

---------- -CAGGACATC CCCCCCGTCC TACTGATGCA TCACCCCCTA

ACTAAGTCAT CCAGGACATC CCCCCCGTCC TACTGATGCA TCACCCCCTA

ACCAAGCCAT CCAGGA---C CTCCCAGTCC CAGTGATGCA TCACCACCTA

CTCAAATTAT CCAGGATATC CTAAAC---- --CTGAGGAA GGAATACTTG

CTCAAATTAT CCAGGATATC CCAAAC---- --CTGATGAA GGAATACTTA

TTATAATTAT ACAGGATATC CTAGTC---- --CCCGTGAA GGAATATTTA

---------- ---------- --CCCAAGCC CAGTGATGAA ACACCACCTA

ACGGT---GC TGAGGGTTTA GGTGCAGGAT ACATCGTGCT CGTCATTGTT

ACGGT---GC TGAGGGTTTA GGTGCAGGAT ACATCGTGCT CGTCATTGTT

AAGAT---GC TGAGAGTTTA GATGGAGGAA TCATCGCTGC AATTGTTGTG

ACAGT---TT GGATGTTTGG GTCATTGCTG TGATTGTTAT TGCCATAGTT

ACAAT---TT GGATGATTGG GTCATTGCTC TGATTGTTAT TGTCATAGTT

GCCAAGAATT AGATGCATGG ATTATTGCTT TGATTGTTAT TACGTCAATT

G--------- TGACACTTCA GGCAAAGGAT ACATTGTTGT TGTGATTGTT

GC---TGTAC TTATTGGCGT TGGATTATTG CTCTGCCTGT ACTGCTGTTT

GC---TGTAC TTATTGGCGT TGGATTATTG CTCTGCCTGT ACTGCTGTTT

GG---CGTCT TAGCTGCCAT TGCAGTAATT G-CTGGTGGT GTATACTTTT

GT---TGGAG TTGCAGTAAT TTGTGTTGTC CCGTACAGAT ATCTTC----

GT---TGCAG TTGCAGTAAT TTGTGTTGCC CTGTACAGAT TTCTTC----

GT---TGGAG TTTTTATACT TTGTCTCATT GTGCTCAGGT GTTTTG----

CTCTGTGTCT CTGCTGGCCT TGTAGTCATT GTCATCGTCG TGTTCGTAGT

TTGCAGACAG AGGAAGAAAG GGAAAGCAGA ATGTAGCGCT ACGTACACCA

TTGCAGACAG AGGAAGAAAG GGAAAGCAGA ATGTAGCGCT ACGTACACCA

TTCATCATAA ATACAACAAG AAAAGGTCGA AGTAA----- ----------

---------- ---------- --AAAGGAGG AAGAAGAAAG GCACATACCT

---------- ---------- --AAGGGAGG AAGAAGAAAG GCACATACCT

---------- ---------- --AGCACAGG AAGAAAACAA ATGTATCTGC

GTATCGACAA AAGAAGAAAG GGAAAAGTGA TATTAGAGCG GAGTACAGTG

CTTATCAGGA TAAAGCAACC ACTGCAACAG AACAGATGAA CTGA

CTTATCAGGA TAAAGCAACC ACTGCAACAG AACAGATGAA CTGA

---------- ---------- ---------- ---------- ----

AACTGATGAG ACCCACAGAG AAGTAAAATT TACTTCTCTC TGA-

AACTGAGGAG AACCACAGAC AAGTAAAATT TACTTCTCTC TGA-

AGCAAGATGA ---------- ---------- ---------- ----

CATACCAGGA TAAATCAGCT ACGACAGCAG AGTAG----- ----

**Figure S4 Comparisons of bovine CD46-TV and other species’ CD46 amino acid sequences.**

Bos_taurus (NP_001229490.1) ; Sus_scrofa (NP_999053.1); human (NP_002380.3); Macaca_mulatta (NP_001182675.1); Mus_musculus (NP_034908.1); Canis_lupus_familiaris (XP_005622378.1)

7 445

Bos_taurus.seq -MRASCTPLK APLRRPERLA SS-GRFAWVL LL--APLLLL PTSSDACDDP

Bos_taurus_CD46-TV.seq -MRASCTPLK APLRRPERLA SS-GRFAWVL LL--APLLLL PTSSDACDDP

Sus_scrofa.seq -MMAFCALRK ALPCRPENPF SS-RCFVEIL WVSLALVFLL PMPSDACDEP

human.seq ---------M EPPGRRECPF PS-WRFPGLL LA--AMVLLL YSFSDACEEP

Macaca_mulatta.seq ---------M APPGRRERPF SS-GRFPGLL LA--TLVLQL SSFSDACEAP

Mus_musculus.seq MTAAPLMPDS THPCRRRKSY TFFWCSLGVY AE--ALLFLL SHLSDACELP

Canis_lupus_familiaris.seq -MTASRAPRT RGPCCPLSPS CS-PRCSQPL RGFLMLLLLH SWVVDACDR-

PRFVSMKPQG TLKPSYSPGE QIVYECRLGF QPVTPGQVLA LVCQDNNTWS

PRFVSMKPQG TLKPSYSPGE QIVYECRLGF QPVTPGQVLA LVCQDNNTWS

PKFESMRPQ- FLNTTYRPGD RVEYECRPGF QPMVPALPTF SVCQDDNTWS

PTFEAMELIG KPKPYYEIGE RVDYKCKKGY FYIPP-LATH TICDRNHTWL

PTFEAMELIG KPKPYYRVGE RVDYKCKKGY FYIPP-LATH TICDRNHTWL

RPFEAMELKG TPKLFYAVGE KIEYKCKKGY LYLSP-YLMI ATCEPNHTWV

PAYISMKPN- VSKMNFDPGD TIFFTCNLGY RPIRPLLPMS SVCQPDNTWT

SLQEG-CKKR RCPTLADPTN GQVILVNGST AFGSEVHYVC NNGYYLLGTN

SLQEG-CKKR RCPTLADPTN GQVILVNGST AFGSEVHYVC NNGYYLLGTN

PLQEA-CRRK ACSNLPDPLN GQVSYPNGDM LFGSKAQFTC NTGFYIIGAE

PVSDDACYRE TCPYIRDPLN GQAVPANGTY EFGYQMHFIC NEGYYLIGEE

PVSDEGCYRE MCPHIRDPLN GEAILANGSY EFGAELHFIC NEGYYLIGKD

PISDAGCIKV QCTMLQDPSF GKVYYIDGSF SWGARAKFTC MEGYYVVGMS

PLKEG-CTKK SCLHPGEPSN GQVVLVDESL LFGSKIQYSC NEGFRLVGQK

ISYCEVSS-- GTGVNWSDNP PTCEKILCQP PPEIQNGKYT NSHKDVFEYN

ISYCEVSS-- GTGVNWSDNP PTCEKILCQP PPEIQNGKYT NSHKDVFEYN

TVYCQVS--- GNVMAWSEPS PLCEKILCKP PGEIPNGKYT NSHKDVFEYN

ILYCELKG-- -SVAIWSGKP PICEKVLCTP PPKIKNGKHT FSEVEVFEYL

ILYCELKD-- -TVAIWSGKP PLCEKILCTP PPKIKNGKHT FSEVEVFEYL

VLHCVLKGD- -DEAYWNGYP PHCEKIYCLP PPKIKNGTHT LTDINVFKYH

NLYCEISSTD SNRVVWSDDP PLCTKILCQP PGKIENGKYS DSHKDEFEYN

EVVTYSCDPS NGPDEYSLVG ESKLTCIGNG EWSSQPPQCK VVKCVYPAIE

EVVTYSCDPS NGPDEYSLVG ESKLTCIGNG EWSSQPPQCK VVKCVYPAIE

EVVTYSCLSS TGPDEFSLVG ESSLFCIGKD EWSSDPPECK VVKCPYPVVP

DAVTYSCDPA PGPDPFSLIG ESTIYCGDNS VWSRAAPECK VVKCRFPVVE

DAVTYSCDPA PGPDPFSLIG ESMIYCGNNS TWSHAAPECK VVKCRFPVVE

EAVSYSCDPT PGPDKFSLVG TSMIFCAGHN TWSNSPPECK VVKCPNPVLQ

EVVTYSCEKS QGTDEYSLIG DNKLICSGDG EWSSNPPECK VVRCPLPDPE

HGTIVSGFGP KYYYKATVVL KCNEGFNLYG NSVVVCGENS TWEPELPKCI

HGTIVSGFGP KYYYKATVVL KCNEGFNLYG NSVVVCGENS TWEPELPKCI

NGEIVSGFGS KFYYKAEVVF KCNAGFTLHG RDTIVCGANS TWEPEMPQCI

NGKQISGFGK KFYYKATVMF ECDKGFYLDG SDTIVCDSNS TWDPPVPKCL

NGKQISGFGK KFYYKATVMF ECDKGYYLNG SDKIVCESNS TWDPPVPKCL

NGRLISGAGE IFSYQSTVMF ECLQGFYMEG SSMVICSANN SWEPSIPKCL

NGKLVLGFSR KYYYKARIEF ECLSGFYHKG TNFAICGSNS TWEPEMPMCL

KVSIPPSTQS PI----PSTQ PPVPSVSVST RSTQHPVPSV SVSTRSTQHP

KVSIPPSTQS PI----PSTQ PPVPSVSVST RSTQHPVPSV SVSTRSTQHP

KDSKP----- --------TD PPAT------ -----PGP-- -------SHP

KVLPPSSTKP PALSHSVSTS STTKSPASSA ---SGPRPT- ------YKPP

KVS------- --------TS PTTKSPTSSA ---SGPRPT- ------YKPP

KGP------- ---------- ---------- ----RPTHP- ------TKPP

KVPIPP---- -------TTS PPILSHTVS- -----SPPS- ------TNSP

IPNVS----- ---------- -GHPPRPTDA SPPNGAEGLG AGYIVLVIVA

IPNVSDSKPT SPTMTSGLSH PGHPPRPTDA SPPNGAEGLG AGYIVLVIVA

G--------- ---------- ---PPSPSDA SPPKDAESLD GGIIAAIVVG

VSNYP----- ---------- --GYPKPEEG ILD----SLD VWVIAVIVIA

VSNYP----- ---------- --GYPKPDEG ILN----NLD DWVIALIVIV

VYNYT----- ---------- --GYPSPREG IFSQ---ELD AWIIALIVIT

IPSVS----- ---------- --GSPKPSDE TPPSDTSGK- GYIVVVIVLC

VLIGVGLLLC LYCCFCRQRK KGKAECSATY TTYQDKATTA TEQMN

VLIGVGLLLC LYCCFCRQRK KGKAECSATY TTYQDKATTA TEQMN

VLAAIAVIAG GVYFFHHKYN KKRSK----- ---------- -----

IVVGVAVICV VPYRYLQRRK KKGTYLTDET HREVKFTSL- -----

IVVAVAVICV ALYRFLQGRK KKGTYLTEEN HRQVKFTSL- -----

SIVGVFILCL IVLRCFEHRK KTNVSAAR-- ---------- -----

VSAGLVVIVI VVFVVYRQKK KGKSDIRAEY SAYQDKSATT AE---
